# Perceptions of a virtual education platform: how plastic surgery education has progressed during the COVID-19 pandemic at one academic institution

**DOI:** 10.1186/s12909-023-04645-y

**Published:** 2023-09-27

**Authors:** Siyou Song, Audrey Nguyen, Micaela Rosser, Gabriela Steiner, Esther A. Kim

**Affiliations:** Department of Surgery, Division of Plastic and Reconstructive Surgery, University of California, San Francisco, 505 Parnassus Avenue, 94143 San Francisco, CA USA

**Keywords:** Medical education, COVID-19, Internship and residency, Education distance, ACGME competency, Medical Knowledge, Interpersonal and communication skills

## Abstract

**Background:**

To continue education during the COVID-19 pandemic, we implemented a Virtual Education Platform (VEP) and Virtual Visiting Professorship (VVP) in March 2020 into our plastic surgery residency curriculum. This study investigated resident and guest speaker perceptions of the VEP since the start of the pandemic.

**Methods:**

The VEP consists of weekly VVP lectures and usual conferences held over Zoom. In May 2020, residents and speakers completed surveys that assessed the perceptions of the VEP using a 5-point Likert scale and open-ended responses. In August 2021, residents also completed follow-up surveys.

**Results:**

A total of 19 (100%) residents and 10 (100%) speakers responded to the 2020 surveys and 15 (88.2%) residents responded to the 2021 follow-up survey. Speakers represented nine academic institutions, one international. 74% of residents responded that they learned a lot or a great deal from the VVP. In 2021, 100% of residents agreed that virtual conferences should remain a core component in PRS residency education, even after social distancing requirements subside.

The VVP lectures were mentioned as the most helpful lectures in both years. Easy accessibility without travel time was the most mentioned advantage of the VEP in both years, with significantly more residents citing this benefit in 2021 (*p* = 0.0076). The most reported disadvantage for residents was the lack of social interaction and community in both years, with significantly more residents in 2021 citing this as a disadvantage (*p* = 0.0307). Residents’ attitudes also shifted such that significantly more residents liked and were satisfied with the VVP lectures from 2020 to 2021 (*p* = 0.04).

**Conclusion:**

Over a year into the COVID-19 pandemic, resident perceptions of a virtual education platform and virtual visiting professorship were very positive. The quick development, implementation, and high efficacy of these educational experiences underscore that learning is possible in alternative forms in unprecedented times.

**Supplementary Information:**

The online version contains supplementary material available at 10.1186/s12909-023-04645-y.

## Background

The COVID-19 pandemic caused by the novel Sars-CoV-2 virus presented an unprecedented disruption to education for plastic surgery residents. Hospitals across the United States suspended elective surgeries, which make up most plastic and reconstructive surgeries. At the University of California San Francisco (UCSF), Tier 1 surgery (Table 1) cancellations began on March 10th, 2020, as the hospital system prepared for a surge of COVID-19 patients. On average, the Division of Plastic and Reconstructive Surgery (DPRS) at UCSF performs 250 surgeries per month at the UCSF campuses. In April 2020, with no Tier 1 surgeries scheduled, this number fell to 62: a 75% decrease. In the authors’ county of San Francisco, shelter-in-place orders were the first in the United States to go into effect on March 17, 2020 [1].  UCSF placed restrictions on group gatherings on March 11, 2020 and transitioned to online platforms for education on March 15, 2020.

**Table 1:** Definitions of Tier classifications

**Tier**	**Definition**	**Examples**
**1**	**Low priority; fully elective;** prognosis not adversely affected by reasonable delay	Benign diseases, surveillance endoscopies, cosmetic procedures, etc.
**2A**	**Intermediate/standard time;** typically scheduled within 3 months in most practice settings	Low risk cancers, stable symptomatic cardiovascular, most spine and orthopedic
**2B**	**Intermediate/time-sensitive;** procedure should be done within one month or prognosis may be impacted	Aggressive cancers, highly symptomatic cardiovascular
**3**	**High priority; life- or limb threatening;** procedure should be done within 7 days or there are likely adverse consequences	Urgent cardiovascular, neurovascular, trauma, some transplants

In response to decreased operative time and social distancing requirements, the Division of Plastic and Reconstructive Surgery immediately created and implemented a Virtual Education Platform (VEP) and a new Virtual Visiting Professorship (VVP) through which experts from around the world were invited as guest lecturers over Zoom. This study evaluates the VEP and VVP and their impact on resident education.

## Methods

### Content of the VEP

At our institution, educational conferences routinely consist of two hours of lecture and one hour of grand rounds on Wednesday mornings, a monthly journal club, and quarterly lectures from visiting professors. The weekly two hours of lecture include morbidity and mortality conference, portfolio conference, research works-in-progress meetings, and lectures based on the American Society of Plastic Surgeons Education Network (EdNet) curriculum [2]. In March 2020, these conferences were transitioned to Zoom (Zoom Inc., San Jose, California) as part of the VEP [3]. Multiple national organizations, including the American Society of Plastic Surgeons (ASPS), American Society for Aesthetic Plastic Surgery (ASAPS), and the American Society for Surgery of the Hand (ASSH) created daily or weekly virtual lectures that were open to resident surgeons across the United States. All residents in the plastic and reconstructive program are required to attend these lectures. The participation rate was 100%, unless residents were excused by the program directors ahead of time for scheduled leave or work hour restrictions. The average participation rate was 78%.

### Development and implementation of the VVP

Given the dearth of elective plastic surgery cases, the VVP was created to supplement the curriculum in time that was previously spent in the operating room. Eleven plastic and reconstructive surgeons and professors were invited to speak as part of the VVP, based on their expertise in subspecialty topics within plastic surgery ranging from hand surgery to microsurgery. These speakers were chosen for their expertise in various subspecialties to meet specific knowledge needs of residents (Table 2). These lectures were given throughout the week in addition to the regularly scheduled lectures. In 2021, the VVP was incorporated into the second hour of the weekly virtual educational conferences as the operative schedules normalized. The goals of these sessions were to continue surgical residency education in the absence of operating time, while also maintaining a sense of community and interactive learning for residents.

**Table 2:** Demographics of guest speakers who completed the speaker survey in 2020. (*n* = 10)

Institution and location	# of speakers	Specialty
USC, California	2	Aesthetic body contouring, microsurgery cerebrovascular reconstruction
UCLA, California	1	Hand
UPenn, Pennsylvania	2	Microsurgery, breast Orthoplastic lower extremity reconstruction
Memorial Sloan Kettering, New York	1	Microsurgery, head and neck reconstruction
Cleveland Clinic, Ohio	1	Microsurgery, abdominal wall reconstruction
Asian Medical Center, South Korea	1	Microsurgery, limb salvage, diabetic limb reconstruction
Northwestern, Illinois	1	Microsurgery, peripheral nerve, TMR, RPNI
Duke, North Carolina	1	Microsurgery, chest wall reconstruction

### Survey development and distribution

To assess the advantages and disadvantages, and progress of the VEP and VVP, we created surveys using the Qualtrics XM online survey platform. The link to the survey and an IRB-approved Information Sheet were emailed to potential participants. All surveys can be found in the supplemental figure.

There were two rounds of surveys. The 2020 surveys consisted of questions for residents and speakers using a five-point Likert scale and open-ended questions. The resident and speaker surveys were sent on May 1, 2020, two months after the initiation of the VEP. The resident survey queried basic demographic information, and the educational experience and impacts of the COVID-19 pandemic on learning. The survey evaluated residents’ perspectives on the effectiveness, advantages, and disadvantages of the VEP and VVP. The guest speaker survey focused on the experience of lecturing through a virtual platform and the impact of online learning on resident education.

The 2021 survey was developed according to the Association for Medical Education in Europe (AMEE) Guide No. 87 guidelines and based on a previously validated questionnaire by Lazaro et al. with a 5-point Likert scale [4, 5]. This survey was administered in August 2021 to Division residents who had participated in the VEP in 2020. The aim was to evaluate the progress of and changing attitudes towards the VEP. Like the 2020 resident survey, the 2021 survey queried the residents’ perspectives on educational experiences during the pandemic, as well as effectiveness, advantages, and disadvantages of the VEP and VVP.

### Data analysis and coding

Averages of each 5-point Likert scale question were calculated for the 2020 and 2021 surveys. The mean scores from the 5-point Likert scale questions for the 2020 and 2021 surveys were compared using Wilcoxon rank and chi-square tests, where appropriate. A *p*-value of less than 0.05 was considered statistically significant. Statistical analyses were done in Microsoft Excel (Microsoft Corporation, 2021) and R (R Core Team, 2021).

The method chosen for the qualitative analysis was conventional qualitative content analysis [6]. In this approach, researchers develop category codes directly from the text. Members of the research team read through all the open-ended course evaluation survey responses and identified codes. After all the responses were coded, the researchers carefully reviewed the information and organized the code into categories. This study was approved by the University of California San Francisco Institutional Review Board.

## Results

### VEP and VVP participants

Each year, there are 21 residents in the Plastic Surgery Residency Program at UCSF. The two residents involved in our study were excluded from taking the surveys to prevent bias. A total of 19 (100%) residents responded to the 2020 survey (10 identified as female, and 9 identified as male). Three residents graduated in 2021, leaving 18 residents who could participate in the second round of surveys. A total of 15 (88%) residents responded to the 2021 survey.

**Fig. 1:**
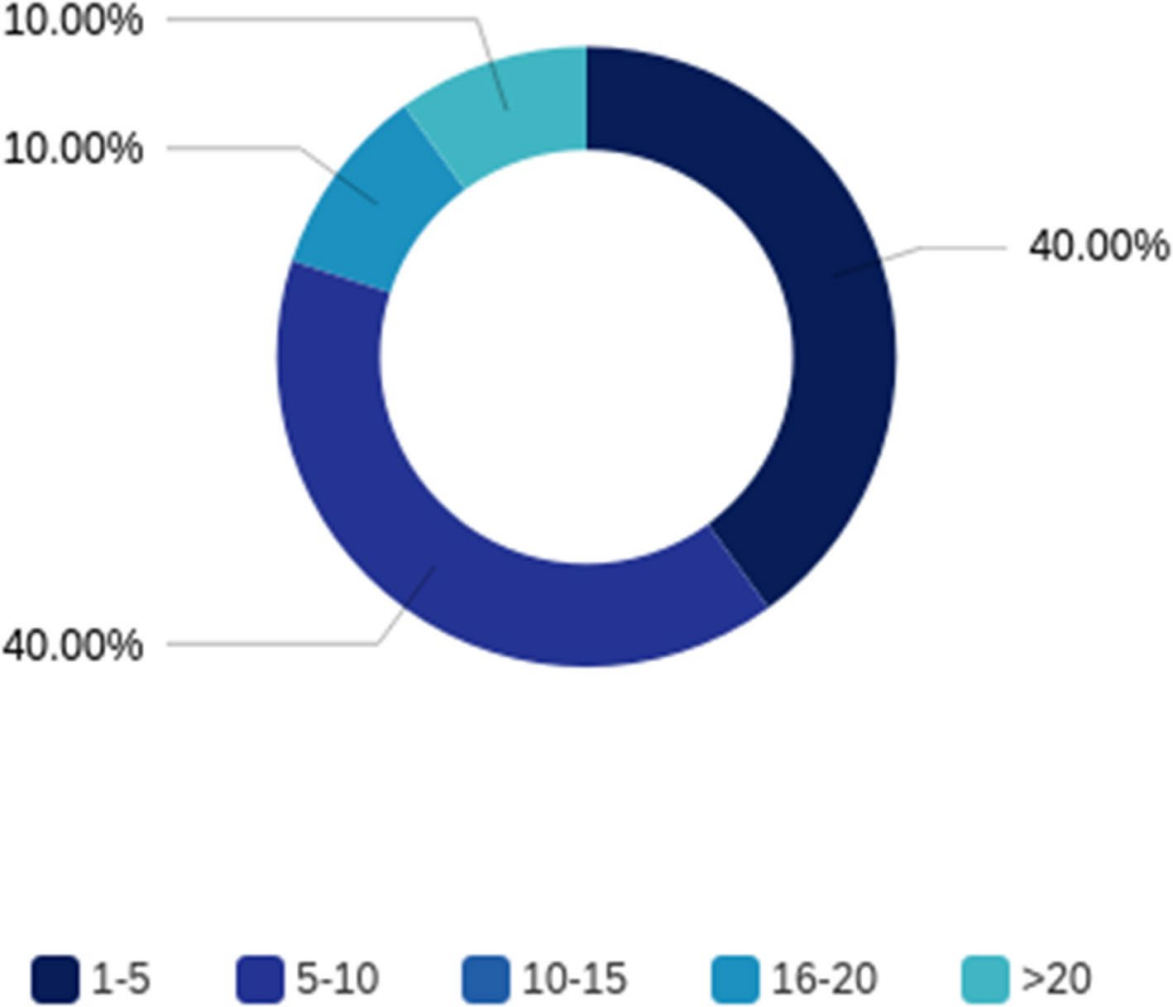
Percentage of guest speakers who gave different numbers of virtual lectures between March to May 2020

From April 10 to May 15, 2020, there were eleven guest speakers in the VVP, ten of whom responded to the 2020 guest speaker survey. All speakers were male and came from various plastic surgery programs including University of Southern California, Memorial Sloan Kettering, University of California Los Angeles, Duke University, Northwestern University, University of Pennsylvania, The Cleveland Clinic, and Asan Medical Center in South Korea (Table 2). The majority (70%) of the speakers specialized in microsurgery (Table 2) covering various reconstructive areas from head and neck, torso to lower extremity. Most speakers had given lectures at other institutions, and most had given between 1 and 10 virtual lectures (Fig. 1).

### Speaker survey responses

**Fig. 2:**
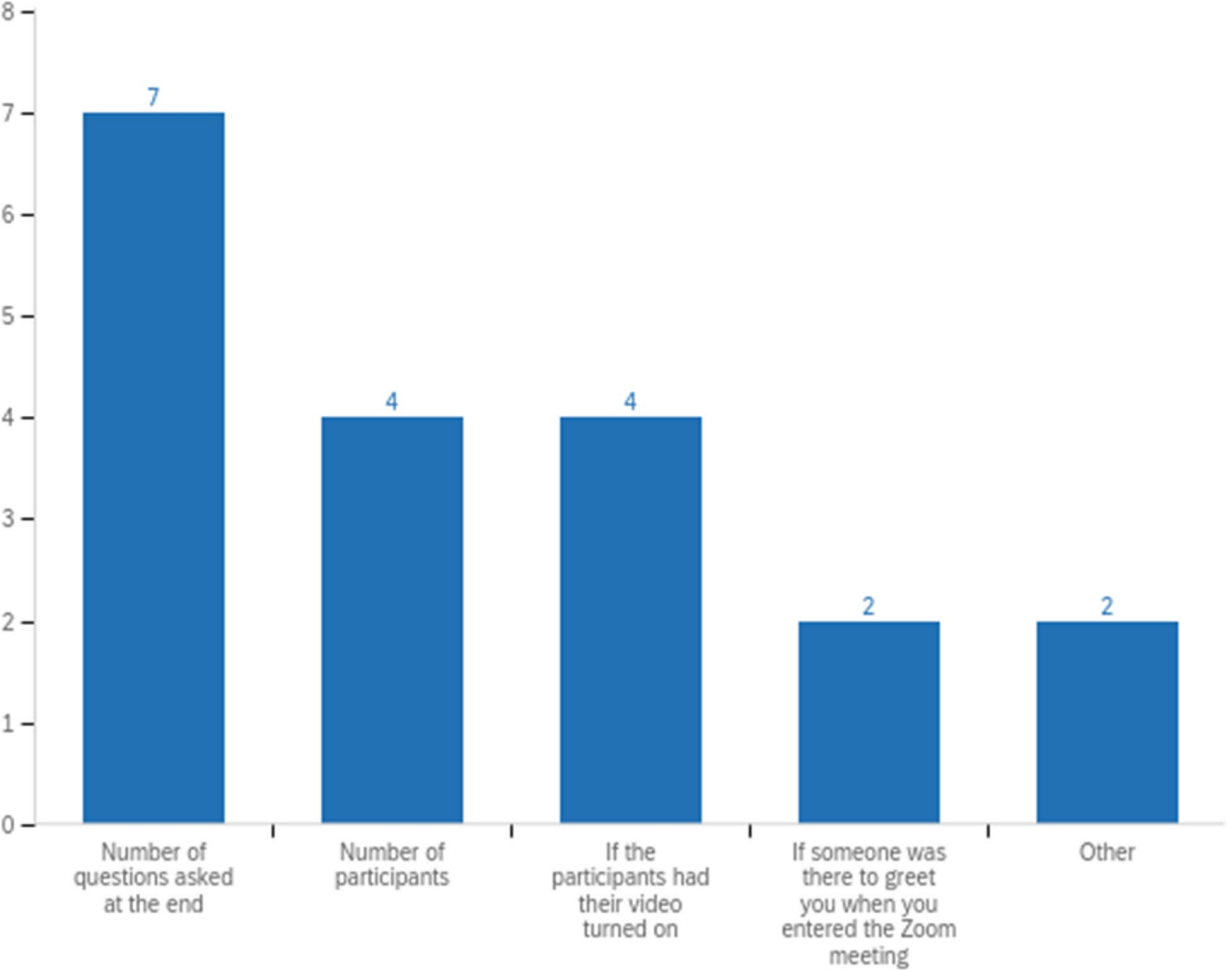
Guest speakers’ opinions about what makes a virtual lecture successful

90% of the 2020 speakers thought that virtual didactic lectures were very important or extremely important to resident education. Half of the speakers responded that it was moderately important to speak to a physical audience. If given the choice, 60% of the speakers prefer in-person lectures. Six speakers liked the Zoom virtual speaker platform a great deal, three liked it somewhat, and only one speaker disliked it somewhat. One speaker summarized the in-person versus virtual lectures well: “I think both approaches can be highly effective, and both serve an important role.” 70% of speakers remarked that the number of questions asked at the end was a marker for a successful virtual lecture (Fig. 2).

### Resident survey responses

**Fig. 3:**
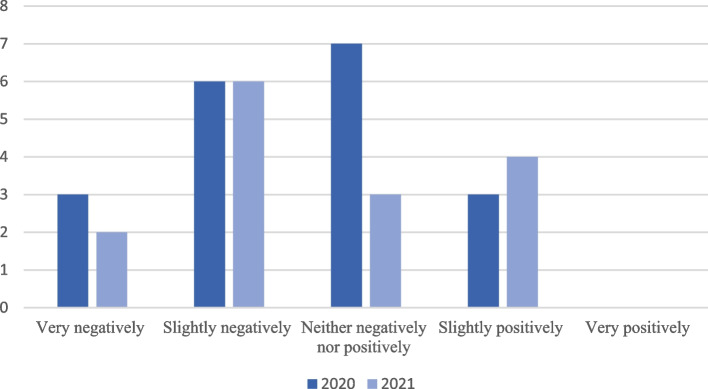
Impact of the COVID-19 pandemic on resident education from 2020 and 2021

When asked how the COVID-19 pandemic affected their education, 9 (47.4%) and 8 (44.4%) residents in 2020 and 2021, respectively, thought their education was negatively impacted. Interestingly, 3 (15.8%) residents in 2020 thought that the pandemic had positively impacted their education and 4 (22.2%) residents thought so in 2021 (Fig. 3). In 2020, 13 (68.4%) residents were concerned about completing their case logs. As surgical cases began to increase later in the pandemic, 14 (83.3%) residents in 2021 reported they were confident in reaching their case log goals. In 2020, 2 (10.5%) residents thought that didactics had an extremely positive impact on their education. In 2021, 5 (27.8%) residents thought that didactics were essential to their education.

Since the start of the VEP, residents’ attitudes shifted such that significantly more residents liked and were satisfied with the VVP lectures in 2021 than in 2020 (*p* = 0.04). The VVP lectures were also mentioned as the most helpful lectures for residents in both years (95% and 47%, respectively). When asked if the lectures should continue even after the shelter-in-place is lifted, 17 (89.5%) residents in 2020 residents responded yes.

When asked about the advantages of the VVP, the free responses fell into three categories: enhanced learning capabilities, tension between VVP and in-person, and improved content material (Table 3). Enhanced learning capabilities is defined as improvements to the existing plastic surgery residency curriculum and included any response that mentioned ability to record lectures, increased faculty attendance, and greater exposure to more guest speakers. One resident responded, “I think it has been so valuable to hear from professors around the world. This time has proven that the virtual lecture really does work, and therefore we don’t have to limit the visiting professorships to a few times a year” (ID#2020-10). Representative quotation from each category are shown in Table 3.

**Table 3:** Distribution of major survey codes and quotes for the advantages of the VVP.

Category	2020 Resident passages (n)	Quotations
Enhanced learning capabilities	9	“Yes, amazing opportunities to learn from experts in specific areas. A great value to the program.” (ID# 2020-5)
Tension between VVP and in-person	4	“Yes. More convenient… no commute stress” (ID# 2020-2) “I actually enjoyed in-person VVP. It was more personal to meet visiting professor(s) face to face.” (ID# 2020-12)
Improved content material	2	“Yes. Good to hear different perspectives and surgical techniques.” (ID# 2020-9)
Total from column (n)	15	

**Fig. 4:**
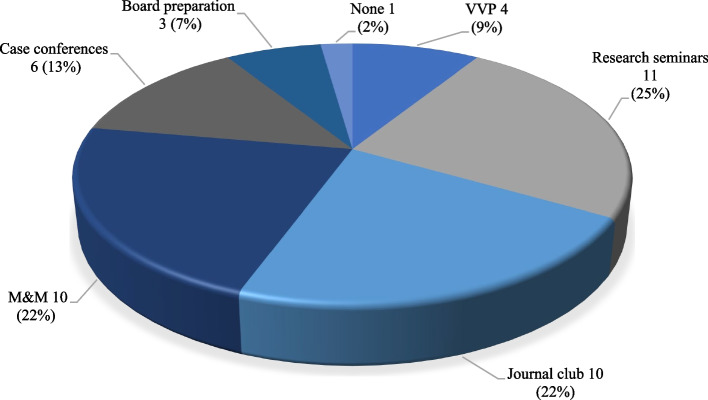
Distribution of 2021 resident responses of the types of in-person conferences that should be completely replaced by virtual conferences. (*n* = 57)

In 2021, 18 (100%) residents agreed that virtual conferences should remain a core component of PRS resident education, even after social distancing requirements subside. Easy accessibility without travel time was the most mentioned advantage for residents in both years (52% and 93%, respectively, *p* = 0.0076). When social distancing is no longer needed, 13 (72.2%) residents thought that 75% or more of the conferences should continue as virtual only. When asked which types of in-person conferences should be completely replaced by virtual conferences, the responses were grand rounds (80%), research seminars (73%), journal club (67%), and M&M conferences (67%) (Fig. 4).

In terms of the advantages of the VEP, review and coding of 81 responses to open-ended survey questions (23 residents from 2020, 15 residents from 2021, and 43 guest speakers from 2020) yielded five major categories: easy accessibility, decreased cost, less time commitment, enhanced learning capabilities, and decreased geographical restraints (Table 4). The easy accessibility category included responses that mentioned decreased travel time and access to a wider audience. Decreased geographical restraints included the ability to learn remotely and without geographical constraints. Enhanced learning capabilities consisted of more lecturers, improved ability to take notes or screenshots, and use of screen sharing when teaching. Representative responses are presented in Table 5.

**Table 4:** Distribution of major survey codes for the advantages of the VEP.

Category	Guest speaker passages (n)	2020 Resident passages (n)	2021 Resident passages (n)	Total from row (n)
Easy accessibility	19	12	14	45
Decreased cost	8	0	0	8
Less time commitment	8	0	0	8
Enhanced learning capabilities	1	10	1	12
Decreased geographical restraints	7	1	0	8
Total from column (n)	43	23	15	81

**Table 5:** Survey responses to the advantages of the VEP.

Category	Quotations
Easy accessibility	“Can participate anywhere even if you’re running late due to clinical duties, traffic, etc.” (ID# 2020-17)
Enhanced learning capabilities	“…now we are recording most lectures so we can have a database of them. I have been wanting to do this for a long time.” (ID #2020-5)
	“Able to concentrate better on topic.” (ID# 2020-12)

The disadvantages of the VEP were also evaluated based on 73 responses (21 residents from 2020, 8 residents from 2021, and 44 guest speakers from 2020. Four major categories emerged: less interaction, increased distraction, screen fatigue, technology issues (Table 6). The category of less interaction included any responses that mentioned a lack of physical connection, less social connection, less human interaction, difficulty assessing audience engagement, and less interaction of speaker with viewers. Sample responses are presented in Table 7. Less interaction was the most mentioned disadvantage of the VEP for residents in both 2020 and 2021, with significantly more residents reporting this disadvantage in 2021 compared to 2020 (*p* = 0.0307).

**Table 6:** Distribution of major survey codes for the disadvantages of the VEP.

Category	Guest speaker passages (n)	2020 Resident passages (n)	2021 Resident passages (n)	Total from row (n)
Less interaction	41	13	7	61
Increased distraction	3	3	1	7
Screen fatigue	0	3	0	3
Technology issues	0	2	0	2
Total from column (n)	44	21	8	73

**Table 7:** Survey responses to the disadvantages of the VEP.

Category	Quotations
Less interaction	“More difficult to connect with the speaker in terms of questions or engage with the rest of the participants.” (ID# 2020-13)
Increased distraction	“Distraction” (ID# 2021-5)
Screen fatigue	“Zoom fatigue” (ID# 2020-3)
Technology issues	“Sometimes poor connection.” (ID# 2020-12)

## Discussion

Although the surgical component of resident education is of paramount importance, the integration of didactic lectures must complement the development of surgical skills. Vast funds of knowledge found on websites, online journal articles, books, and videos can be accessed from the comfort of one’s own home with as little as a smartphone. As technology becomes more advanced, we have seen its increasing footprint in daily practice, from online resources and videos to telehealth [7, 8]. It is not surprising, therefore, that the COVID-19 pandemic was the catalyst that drove the transition to online didactics for our plastic surgery residency program.

Of course, online surgical education has existed for many years, mostly in the setting of pre-recorded videos. For example, Lee et al. developed the microsurgical online education platform at Stanford Medical Center to address the shortcomings of traditional education [9]. However, the effect of a fully virtual curriculum on the satisfaction and perceptions of plastic surgery residents education over multiple timepoints has not been studied. We therefore examined resident perspectives about the VEP at the beginning of the COVID-19 pandemic and how those perspectives have changed as the VEP has become integrated into the educational experience over the past year. Our study shows that adaptation to a new form of didactic learning is pivotal in continuing resident education, even after the COVID-19 shelter-in-place orders are fully lifted. The consensus among respondents to our 2020 and 2021 surveys was that the adaptability of the UCSF Plastic Surgery Program and the change to a virtual platform has been valuable despite the ongoing pandemic. All residents in 2021 agreed that virtual conferences should remain a core component of their residency education, even after the pandemic ends.

There was overwhelming positive feedback for the UCSF VEP and VVP. The virtual platform provided several advantages over in-person learning, including more lectures from international speakers, greater accessibility with less to no travel time, and greater diversity of educational content. In fact, 73% of residents remarked that at least 75% of the in-person meetings should be moved online. These advantages of virtual education continue to persist, as noted in other studies that report virtual learning during the pandemic has provided additional time for self-study with less concern about commuting and parking [10, 11].

The VEP had its own challenges, while also creating opportunities that had not been considered before in resident education. Online learning can reach a wider audience, but often at the expense of a less personal experience. The most mentioned disadvantage of the VEP was a lack of human interaction and social connection, with significantly more residents citing this disadvantage in 2021 than in 2020. The guest speakers also agreed that the lack of interpersonal connection was a disadvantage, and they would not choose a virtual lecture over an in-person lecture. Foad Nahai describes a “hunger for that face-to-face contact” in his editorial discussing the future of plastic surgery meetings [12]. Other challenges include access to reliable internet and ease of use for those less technologically facile [13]. Despite these limitations, the VEP enabled new ways of teaching with unique advantages, including greater accessibility to learning materials and connections with experts around the world.

Although most residents in both years of the survey responded that the COVID-19 pandemic has negatively impacted their education, the proportion who felt that the pandemic had impacted their education in a positive way still increased between 2020 and 2021. This may be partly explained by the VEP, which allowed for the preservation of the 15 h of teaching per month for residents. Another contributing factor may be that the VEP is a dynamic educational tool that allows surgical educators to incorporate feedback and improve the VEP’s learning experience for trainees, as evidenced by significantly more residents being satisfied with the VVP in 2021 than in 2020.

The proportion of residents who thought didactics were very important or essential to their education increased between 2020 and 2021, and not surprisingly, all residents reported operative time as a crucial part of surgical education. Clearly, didactics and the VEP can supplement deficiencies caused by social distancing requirements but cannot replace operative time. However, interactive virtual lectures, surgical videos, and case examples can be a powerful adjunct to resident education. The importance of surgical videos to learning by bringing a live perspective and thought-process of the narrating surgeon is one of the reasons that *PRS* introduced the Video Plus article [14].

There are limitations to this study, including its single-institution survey-based design and small sample size. Residents may also have recall bias when filling out the surveys. Additionally, given the abrupt onset of the COVID-19 Pandemic, many presenters were asked to transition their face-to-face lectures to a virtual platform. At the start of the COVID-19 Pandemic, presenters may not have been as experienced with virtual lecturing, which could have impacted their online delivery and resident learning. However, this study examines a novel framework for resident education that continues to have high satisfaction in an unprecedented time in education and healthcare. As the pressure has been placed on programs to decrease work hours in the hospital by guidelines from the American College of Graduate Medical Education (ACGME), the result has been greater reliance on individual learning accessed online [15]. The “future of plastic surgery education” is evolving into this combination of didactic, formal lectures from experts in the field in a digital world. With further advancements, virtual operative theater lectures may happen in the foreseeable future. Future studies should focus on multi-institution surveys to understand the risks and benefits of virtual plastic surgery education more broadly and to evaluate how a sense of human connection and social interaction can be preserved with virtual education. We also hope to provider longitudinal surveys in the future to observe the trends of virtual learning over time.

## Conclusion

Our study shows that over a year into the COVID-19 pandemic, resident perceptions of a virtual education platform and virtual visiting professorship were very positive. The quick development, implementation, and high efficacy of these virtual educational experiences during an unprecedented time in healthcare underscore that learning can still be achieved in alternative forms in unprecedented times. Virtual education has become an integral part of the PRS education that will persist well after the end of the pandemic. Due to the overall positive feedback from this study, our institution will continue this educational framework for resident learning even after the COVID-19 Pandemic subsides. We recommend that other plastic surgery residency programs appreciate the importance and continuation of virtual learning since the COVID-19 Pandemic. Our VEP experience elucidated the positive experience that most residents experienced with virtual learning; the major advantages of the VEP (e.g., easy accessibility, decreased travel time); and the importance of social interaction and community in residency education.

### Supplementary Information

**Additional file 1:**  **Supplemental Figure 1.** Guest speaker survey to assess for perceptions of the VVP in 2020. **Supplementary Figure 2.** Resident survey to assess the perceptions of the VVP in 2020. **Supplemental Figure 3.** Resident survey to assess the perceptions of the VVP in 2021.

## Data Availability

The datasets used and/or analyzed during this study are available from the corresponding author on reasonable request.

## Abbreviations

VEP: Virtual education platform
VVP: Virtual visiting professorship
PRS: Plastic and reconstructive surgery
