# Supplementary material for: Perceptions of a virtual education platform: how plastic surgery education has progressed during the COVID-19 pandemic at one academic institution
Source: BMC Med Educ. 2023 Sep 27;23:708. doi: 10.1186/s12909-023-04645-y (PMC10537510; doi:10.1186/s12909-023-04645-y)
Supplement: Supplementary file 1 — Additional file 1: Supplemental Figure 1. Guest speaker survey to assess for perceptions of the VVP in 2020. Supplementary Figure 2. Resident survey to assess the perceptions of the VVP in 2020. Supplemental Figure 3. Resident survey to assess the perceptions of the VVP in 2021. [file 12909_2023_4645_MOESM1_ESM.docx]

**Supplemental figures for the VVP manuscript**

**Supplemental figure 1. Guest speaker survey to assess for perceptions of the VVP in 2020.**

UCSF Plastic Surgery Virtual Visiting Professor Survey

Name

________________________________________________________________

What institution are you from?

________________________________________________________________

What is your specialty within plastic surgery?

________________________________________________________________

What is your gender?

- Male (1)
- Female (2)
- Prefer not to say (3)

How many virtual lectures have you given during shelter-in-place because of the COVID-19 pandemic?

- 1-5 (1)
- 5-10 (2)
- 10-15 (3)
- 16-20 (4)
- >20 (5)

How important do you think virtual lectures are to resident education?

- Extremely important (1)
- Very important (2)
- Moderately important (3)
- Slightly important (4)
- Not at all important (5)

How important is it to speak to a physical audience versus a virtual audience?

- Extremely important (1)
- Very important (2)
- Moderately important (3)
- Slightly important (4)
- Not at all important (5)

If you had the choice, would you rather give a/an:

- Virtual Lecture (1)
- In-person Lecture (2)
- Neither (3)
- Both (4)

Please explain your answer to the above question:

________________________________________________________________

How much did you like giving a virtual lecture through the Zoom platform?

- Like a great deal (1)
- Like somewhat (2)
- Neither like nor dislike (3)
- Dislike somewhat (4)
- Dislike a great deal (5)

What makes a virtual lecture successful?

- Number of participants (1)
- Number of questions asked at the end (2)
- If the participants had their video turned on (3)
- If someone was there to greet you when you entered the Zoom meeting (4)
- Other (5) ________________________________________________

What are the advantages of giving a virtual lecture (you may choose more than one)?

- No Travel Time (1)
- Cost (2)
- Less time committment (3)
- Access to wider audience (4)
- Geography (5)
- Other (6) ________________________________________________

What are the disadvantages of giving a virtual lecture (you may choose more than one)?

- Lack of physical connection (1)
- Less social connection (2)
- Lack of physical audience (3)
- Difficulty in assessing audience engagement (4)
- Less interactive (5)
- Less formal (6)
- More distractions (7)
- Other (8) ________________________________________________

What made you agree to give a virtual lecture?

________________________________________________________________

Any additional comments?

________________________________________________________________

**Supplemental figure 2. Resident survey to assess the perceptions of the VVP in 2020.**

UCSF Plastic Surgery Virtual Education Survey

Name

________________________________________________________________

Email address

________________________________________________________________

What is your year in residency?

- R1 (1)
- R2 (2)
- R3 (3)
- Research Resident (4)
- R4 (5)
- R5 (6)
- R6 (7)

What is your gender?

- Male (1)
- Female (2)
- Other (3)
- Prefer not to say (4)

How do you think the COVID-19 pandemic has affected your education?

- Very positively (1)
- Somewhat positively (2)
- Neutral (3)
- Somewhat negatively (4)
- Very negatively (5)

In general, how much do you think the following contribute to your plastic surgery education?

|  | Extremely positive (1) | Somewhat positive (2) | Neither positive nor negative (3) | Somewhat negative (4) | Extremely negative (5) |
| --- | --- | --- | --- | --- | --- |
| In-person didactic lectures (1) |  |  |  |  |  |
| Virtual didactic lectures (2) |  |  |  |  |  |
| Operating time (3) |  |  |  |  |  |
| Being the consult resident (4) |  |  |  |  |  |
| Caring for inpatients (5) |  |  |  |  |  |
| Managing an inpatient team (6) |  |  |  |  |  |
| Taking Max/face call (7) |  |  |  |  |  |
| Taking hand call (8) |  |  |  |  |  |
| Oral Boards Review (9) |  |  |  |  |  |
| In-service and Written Boards review (10) |  |  |  |  |  |
| Journal club (11) |  |  |  |  |  |
| Clinical Service Conference (12) |  |  |  |  |  |
| Portfolio Conference (13) |  |  |  |  |  |
| Research Meetings (14) |  |  |  |  |  |

During this COVID-19 pandemic, how anxious or worried are you about:

|  | A great deal (1) | A lot (2) | A moderate amount (3) | A little (4) | None at all (5) |
| --- | --- | --- | --- | --- | --- |
| Your surgical education (1) |  |  |  |  |  |
| Your clinical experience (2) |  |  |  |  |  |
| Your operative experience (3) |  |  |  |  |  |
| Completing your case log goals (4) |  |  |  |  |  |

Regarding the UCSF Virtual Visiting Professor (VVP) lecture series on Zoom:

|  | A great deal (1) | A lot (2) | A moderate amount (3) | A little (4) | None at all (5) |
| --- | --- | --- | --- | --- | --- |
| How much did you like it? (1) |  |  |  |  |  |
| How much has it helped your education? (2) |  |  |  |  |  |
| How much did you learn? (3) |  |  |  |  |  |
| How much do you think it positively affected your development as a plastic surgeon? (4) |  |  |  |  |  |

Regarding all of the UCSF ZOOM Academic Events (i.e. journal club, M&M, portfolio, etc.):

|  | A great deal (1) | A lot (2) | A moderate amount (3) | A little (4) | None at all (5) |
| --- | --- | --- | --- | --- | --- |
| How much did you like it? (1) |  |  |  |  |  |
| How much has it helped your education? (2) |  |  |  |  |  |
| How much did you learn? (3) |  |  |  |  |  |
| How much do you think it has positively affected your development as a plastic surgeon? (4) |  |  |  |  |  |

Before the COVID-19 pandemic and shelter-in-place orders, how important did you think virtual didactic lectures were to your education?

- Extremely important (1)
- Very important (2)
- Moderately important (3)
- Slightly important (4)
- Not at all important (5)

After the move to a virtual education platform because of shelter-in-place, how important do you think virtual didactic lectures are to your education?

- Extremely important (1)
- Very important (2)
- Moderately important (3)
- Slightly important (4)
- Not at all important (5)

How often did you attend the following virtual lectures:

|  | Always (1) | Most of the time (2) | About half the time (3) | Sometimes (4) | Never (5) |
| --- | --- | --- | --- | --- | --- |
| UCSF Virtual Visiting Professor (1) |  |  |  |  |  |
| UCSF Plastic Surgery attendings (2) |  |  |  |  |  |
| UCSF attendings from other departments (3) |  |  |  |  |  |
| UCSF Oral Boards Review (4) |  |  |  |  |  |
| ASPS Virtual Grand Rounds (5) |  |  |  |  |  |
| Aesthetic Society (6) |  |  |  |  |  |
| Allergan-sponsored (7) |  |  |  |  |  |
| AO CMF (8) |  |  |  |  |  |
| ZO Skin Care Lecture Series (9) |  |  |  |  |  |
| ASSH (10) |  |  |  |  |  |
| BSCOSO (11) |  |  |  |  |  |
| KLS Martin (12) |  |  |  |  |  |
| Mentor-sponsored (13) |  |  |  |  |  |
| Other plastic surgery programs (U Michigan, Duke, UCSD, etc) (14) |  |  |  |  |  |

If applicable to you, what were the reasons why you did not attend the UCSF Virtual Visiting Professor Lecture Series?

- Clinical service demands (1)
- Operating (2)
- Did not know about them (3)
- Did not know how to sign in to the Zoom platform (4)
- Vacation/Day Off (5)
- Forgot (7)
- Other (6) ________________________________________________

If applicable to you, what were the reasons why you did not attend the UCSF Zoom Academic Conferences (i.e. journal club, clinical service conference, portfolio, etc)?

- Clinical service demands (1)
- Operating (2)
- Did not know about them (3)
- Did not know how to sign in to the Zoom platform (4)
- Vacation/Day off (5)
- Forgot (7)
- Other (6) ________________________________________________

If applicable to you, what were the reasons why you did not attend the non-UCSF virtual lectures?

- Not interested in the topic (1)
- Too many to choose from (2)
- Clinical service demands (3)
- Operating (4)
- Did not know about them (5)
- Did not know how to access them or log in (6)
- Vacation/Day off (7)
- Zoom fatigue (8)
- Other (9) ________________________________________________

Which Zoom lectures and/or meetings did you find the most helpful to your education (you may choose multiple answers, but no more than 3)?

- UCSF Virtual Visiting Professor (1)
- Clinical Service Conference (2)
- Portfolio (3)
- Resident Teaching Conference (4)
- Oral Boards Review (5)
- Journal Club (6)
- Research Meeting (7)
- ASPS Virtual Grand Rounds (8)
- Other virtual lectures (9) ________________________________________________

What was your favorite UCSF Zoom lecture and why?

________________________________________________________________

What are the advantages of Zoom or virtual education?

________________________________________________________________

What are the disadvantages of Zoom or virtual education?

________________________________________________________________

What was your favorite UCSF Zoom lecture and why?

________________________________________________________________

What was your favorite non-UCSF virtual lecture and why?

________________________________________________________________

Do you think we should continue UCSF Virtual Visiting Professor lectures after the shelter-in-place is lifted? Why or why not?

________________________________________________________________

What other UCSF Plastic Surgery academic conferences do you think we should continue via Zoom or other virtual platform?

- Clinical Service Conference (1)
- Portfolio (2)
- Journal Club (3)
- Resident Teaching Conference (4)
- Research Meeting (5)
- None (6)

If you chose an answer for the above question, why would you like to continue those via Zoom or other virtual platform?

________________________________________________________________

**Supplemental figure 3. Resident survey to assess the perceptions of the VVP in 2021.**

**Demographics:**

What is your current age (options in years)?

- 20-25
- 26-30
- 31-35
- 36-40
- 40+

Which option best describes your current level of plastic and reconstructive surgical training?

- PGY1 resident
- PGY2 resident
- PGY3 resident
- PGY4 resident
- PGY5 resident
- PGY6 resident
- PGY7 resident

**COVID-19 Pandemic impacts:**

How has the COVID-19 pandemic impacted your surgical education?

- Very negatively
- Slightly negatively
- Neither negatively nor positively
- Slightly positively
- Very positively

How important are didactic lectures in your surgical education?

- Not important
- Slightly important
- Moderately important
- Quite important
- Essential

How important is operative time in your surgical education?

- Not important
- Slightly important
- Moderately important
- Quite important
- Essential

How confident are you in reaching your case log goals?

- Not at all confident
- Slightly confident
- Moderately confident
- Quite confident
- Extremely confident

**Virtual Visiting Professorship (VVP) lectures:**

How satisfied are you with the VVP lectures?

- Not at all satisfied
- Slightly satisfied
- Moderately satisfied
- Quite satisfied
- Extremely satisfied

How much did you learn from the VVP lectures?

- None
- Minimal amount of learning
- Moderate amount of learning
- Quite amount of learning
- A lot of learning

How should the majority of VVP lectures be administered in the future?

- Online (i.e. Zoom)
- In-person

**Virtual versus traditional in-person conferences**.

Given your experience with virtual education opportunities, rate how likely you are to agree with each statement:

Virtual conferences can replace in-person conferences for some lecture formats.

| Strongly disagree | Disagree | Neutral | Agree | Strongly Agree |
| --- | --- | --- | --- | --- |

Case conferences (i.e., lectures based on case presentations) are better done virtually than in-person.

| Strongly disagree | Disagree | Neutral | Agree | Strongly Agree |
| --- | --- | --- | --- | --- |

Department grand rounds (i.e., lectures from department faculty) are better done virtually than in-person.

| Strongly disagree | Disagree | Neutral | Agree | Strongly Agree |
| --- | --- | --- | --- | --- |

Visiting professorships (i.e. lectures from visiting faculty from other institutions) are better done virtually than in-person.

| Strongly disagree | Disagree | Neutral | Agree | Strongly Agree |
| --- | --- | --- | --- | --- |

Didactics curriculum are better done virtually than in-person.

| Strongly disagree | Disagree | Neutral | Agree | Strongly Agree |
| --- | --- | --- | --- | --- |

Morbidity and mortality conferences are better done virtually than in-person.

| Strongly disagree | Disagree | Neutral | Agree | Strongly Agree |
| --- | --- | --- | --- | --- |

Journal clubs are better done virtually than in-person.

| Strongly disagree | Disagree | Neutral | Agree | Strongly Agree |
| --- | --- | --- | --- | --- |

Notwithstanding current social distancing needs, in-person conferences are still preferred for all conference formats.

| Strongly disagree | Disagree | Neutral | Agree | Strongly Agree |
| --- | --- | --- | --- | --- |

Virtual conferences should remain a core medium in PRS residency education even after social distancing requirements end.

| Strongly disagree | Disagree | Neutral | Agree | Strongly Agree |
| --- | --- | --- | --- | --- |

**Future directions of virtual learning:**

How likely are you to continue using virtual conferencing to supplement your residency education in the future?

- Very unlikely
- Unlikely
- Likely
- Very likely
- Unsure

Once social distancing is no longer needed, what proportion of conferences should be virtual only?

- 0%
- 25%
- 50%
- 75%
- 100%

Which type(s) of in-person conference(s) should be **partially** replaced by virtual conferences? Select all that apply.

- Case conferences
- Grand rounds
- Visiting professor lectures
- Research seminars
- Board preparation
- Morbidity & mortality
- Journal club
- None

Which type(s) of in-person conference(s) should be **completely** replaced by virtual conferences? Select all that apply.

- Case conferences
- Grand rounds
- Visiting professor lectures
- Research seminars
- Board preparation
- Morbidity & mortality
- Journal club
- None

How can we improve virtual learning opportunities in the future?_________________________

**Zoom use:**

How many times per conference do you experience difficulties with the conferencing technology (e.g. slow connection, lost connection, audio or visual issues)?

- 0
- 1-2
- 3-4
- 5-6
- >6

Which Zoom lectures did you find the most helpful? Select all that apply.

- Case conferences
- Grand rounds
- Visiting professor lectures
- Research seminars
- Board preparation
- Morbidity & mortality
- Journal club
- None

Compared to in-person conferences, what are the **advantages** of Zoom educational conferences?

- No travel time
- Cost
- Less time commitment
- Access to wider audience
- Geography
- Other

If you selected "other," please specify:_______________________________________________

Compared to in-person conferences, what are the **disadvantages/ barriers** of Zoom educational conferences?

- No travel time
- Cost
- Less time commitment
- Access to wider audience
- Geography
- Other

If you selected "other," please specify:_______________________________________________
